# Stressors and resources mediate the association of socioeconomic position with health behaviours

**DOI:** 10.1186/1471-2458-11-798

**Published:** 2011-10-13

**Authors:** Bob C Mulder, Marijn de Bruin, Hanneke Schreurs, Erik JC van Ameijden, Cees MJ van Woerkum

**Affiliations:** 1 Communication Science, Wageningen University, Hollandseweg 1, 6706 KN, Wageningen, The Netherlands; 2 Municipal Health Service Utrecht, Jaarbeursplein 17, 3521 AN, Utrecht, The Netherlands

## Abstract

**Background:**

Variability in health behaviours is an important cause of socioeconomic health disparities. Socioeconomic differences in health behaviours are poorly understood. Previous studies have examined whether (single) stressors or psychosocial resources mediate the relationship between socioeconomic position and health or mortality. This study examined: 1) whether the presence of stressors and the absence of resources can be represented by a single underlying factor, and co-occur among those with lower education, 2) whether stressors and resources mediated the relation between education and health behaviours, and 3) addressed the question whether an aggregate measure of stressors and resources has an added effect over the use of individual measures.

**Methods:**

Questionnaire data on sociodemographic variables, stressors, resources, and health behaviours were collected cross-sectionally among inhabitants (*n *= 3050) of a medium-sized Dutch city (Utrecht). Descriptive statistics and bootstrap analyses for multiple-mediator effects were used to examine the role of stressors and resources in mediating educational associations with health behaviours.

**Results:**

Higher levels of stressors and lower levels of resources could be represented by a single underlying factor, and co-occurred among those with lower educational levels. Stressors and resources partially mediated the relationship between education and four health- behaviours (exercise, breakfast frequency, vegetable consumption and smoking). Financial stress and poor perceived health status were mediating stressors, and social support a strong mediating resource. An aggregate measure of the stressors and resources showed similar associations with health behaviours compared to the summed individual measures.

**Conclusions:**

Lower educated groups are simultaneously affected by the presence of various stressors and absence of multiple resources, which partially explain socioeconomic differences in health behaviours. Compared to the direct associations of stressors and resources with health behaviours, the association with socioeconomic status was modest. Therefore, besides addressing structural inequalities, interventions promoting financial management, coping with chronic disease, and social skills training have the potential to benefit large parts of the population, most notably the lower educated. Further research is needed to clarify how stressors and resources impact health behaviours, why this differs between behaviours and how these disparities could be alleviated.

## Background

Indicators of socioeconomic position, such as education, occupation, income and wealth, are negatively related to morbidity and mortality [1-8]. In order to intervene in these disparities, it is important to understand how lower socioeconomic position leads to increased morbidity and mortality. Previous research suggests that an important cause lies in the higher prevalence of risky behaviours such as smoking, drinking, physical inactivity and unhealthy dietary habits [9-19]. But then, how does socioeconomic position translate into differences in health behaviours? The observation that socioeconomic position is negatively correlated with morbidity, mortality and health behaviours suggests that there is a set of common, general determinants of health behaviours that is related to socioeconomic position. A perusal of the literature suggests that stressors, such as financial stress and psychological distress [e.g., [20,21]], and a lack of psychosocial resources such as perceived life control [e.g., 22] may group among those with a lower socioeconomic status. Moreover, many studies have found an impact of stress [20,23-27] and resources [26,28-36] on morbidity and mortality. Hence, stressors and resources may be good candidates when looking for general determinants that explain how socioeconomic status translates into health behaviours. For example, financial stress may lead to feelings of anxiety and depression [20,25,29]. In turn, anxiety and depression have been found to predict smoking and waist circumference [20]. Over time, worsening physical and mental health as a result of stress and unhealthy lifestyle may thus become additional stressors themselves. Therefore, stressors under scrutiny in the present article are financial stress, poor physical health and psychological distress.

At the other side of the balance a reserve capacity of several resources such as perceived control (i.e. mastery), social cohesion and social support may positively impact health behaviours [26,28-31]. Perceived control is an important resource for coping with stress, because the belief that one has a certain degree of control over the outcomes in one's life determines emotional and behavioural responses to negative events [e.g., [28,37]]. It has indeed been shown that lower education is associated with lower scores on measures of control, and that, in turn, these are related to either worse health or unhealthy behaviours [20,22,35,36,38]. Similarly, individual social support and neighbourhood social cohesion are resources that vary with measures of socioeconomic position, and that provide tangible, emotional or informational support when dealing with problems [28,30,31]. Hence, perceived life control, social support and neighbourhood cohesion are the psychosocial resources examined in the present article.

Although many studies have looked at the impact of stress and resources in relation to morbidity and mortality, fewer studies examine their impact on health behaviours [38-40]. Moreover, these studies have typically focused on either a single stressor or a single resource, while it is likely that the absence of multiple resources and the presence of multiple stressors co-occur among the lower educated. Other studies have combined stressors and resources into one measure, leaving questions as to what extent specific factors contribute to health disparities, or whether such an aggregate measure can be preferred above examining the specific effects of individual mediators [28,41]. The objectives of the present study are therefore, first, to examine whether high levels of stressors and a lack of resources co-occur among the lower educated. A related objective is to examine whether stressors and resources can be represented by a single underlying factor, as is expected, because the absence of a resource such as life control can well be considered a stressor; and second, to examine whether the relation between educational level and four health behaviours (i.e., exercise, vegetable consumption, breakfast frequency and smoking) is mediated by stressors and resources simultaneously. The final aim is to examine whether an aggregate measure of stressors and resources has stronger associations with the health behaviours than the sum of the individual associations, as has been suggested but, to our knowledge, has not been tested empirically [28].

## Methods

### Study design and sample

In 2008, cross-sectional data were collected in the Dutch city of Utrecht using the Health Survey (HS). The HS consists of a self-administered questionnaire which is distributed every 2 or 3 years among a sample of the city population of 16 years and older. This sample is stratified according to neighbourhood of residence. Inhabitants (n = 7500) were approached by postal mail to participate in the survey, 2413 (32.4%) of whom returned the filled-out questionnaires within two weeks. After two weeks, non-respondents were contacted by telephone providing an additional 787 respondents (10.1%). Another two weeks later, remaining non-responders were contacted personally at their home address to prompt them to return the filled-out questionnaire, yielding the final 649 (8.7%) respondents. This resulted in a total of 3916 respondents (response rate 52.2%; including 67 respondents for whom it was not registered at what step their questionnaire was included). The present study is based on a secondary analysis of these data.

### Measures

Educational level was used as an indicator for socioeconomic position [42,43], and respondents whose main occupation was studying (n = 419, 10.7%) were omitted from the analysis, since they had not yet achieved their final education level. Educational attainment was categorized in four levels: 1) no education and primary school, 2) lower vocational school and intermediate secondary school, 3) intermediate vocational school and higher secondary school, and 4) higher vocational school and university.

Three stressors and three resources were measured. First, financial stress was measured with two items: 1) 'Have you had any difficulty getting by on the household income?' (1 = 'No difficulty whatsoever', 4 = 'Great difficulty'), and 2) 'How is the current financial situation of the household?' (1 = 'Have to go into debt', 5 = 'Still have a lot of money left'). Both items correlated satisfactorily (*r *= .65), corresponding with a Cronbach's alpha of .79. Second, suboptimal physical health was included as a stressor. Since people cope differently with disease [44], rather than using the absence/presence of chronic disease as a stressor, we used perceived health status. This was measured with the single validated item 'How would you rate your health in general?' (1 = 'excellent', 5 = 'poor') [45]. Chronic disease itself was treated as a confounder rather than a stressor, since it may directly cause differences in health behaviours, for instance, through disability. Third, psychological distress was measured with the 10-item Kessler Psychological Distress Scale (Cronbach's α = .92) [46]. Although psychological distress could be both a stressor or an indicator of stress, it is argued - similar to perceived health status - that psychological distress is an indicator of how stressed someone is by their circumstances, and this may vary across individuals in similar circumstances [20].

We also measured three resources. Perceived life control was measured with the Pearlin & Schooler Mastery Scale (Cronbach's α = .83) [47]. Examples of items are 'I have little control over the things that happen to me' or 'Whatever happens in the future largely depends on myself'. All 7 items are scored on a 5-point scale from 'totally agree' to 'totally disagree'. The second resource was perceived social support, measured with 11 items on a 3-point scale ('yes', 'more or less', 'no'; Cronbach's α = .89). Examples of items are 'I have a lot of people I can trust completely' and 'When I feel the need, I can always contact my friends'. Third, social cohesion in the neighbourhood was measured with 5 items on a 5-point scale (1 = 'totally agree', 5 = 'totally disagree'), such as 'The people in my neighbourhood help each other' (Cronbach's α = .81).

All behavioural measurements were self-reported. Exercise was measured in minutes per week by asking participants to indicate the typical number of exercise days per week during the last few months and the average duration of exercise on such a day. Vegetable consumption was expressed in serving spoons per day, and measured by asking how many days in the week they normally ate boiled, fried or raw vegetables and salads, and the number of serving spoons they normally consumed on such a day. Breakfast frequency was added since many studies have found an inverse association with obesity and chronic disease, which may be explained by several mechanisms, such as through metabolic pathways that help control appetite throughout the day [48]. Breakfast frequency was assessed with one item: 'How many days a week do you usually eat breakfast?'. For smoking, people were asked to report the daily number of cigarettes and weekly number of cigars they typically smoke. Demographic characteristics such as age, gender and ethnicity, were also measured. Finally, respondents reported whether they suffered from cardiovascular diseases, lung diseases, musculoskeletal disorders, cancer or diabetes.

### Statistical analysis

For all stressor and resource scales (mediators) the mean item score was calculated. The original response scales varied in ranges, which therefore had to be adjusted to enable comparison. All scales were thus converted to the smallest range of any of the mediators, which was 1 to 3 for social support. For all resulting scales, higher scores indicated higher levels of the particular stressor or resource.

First, bivariate correlations were computed to explore whether educational level, mediators and health behaviours were associated in the expected directions. Next, co-occurrence of stressors and mediators within individuals was examined by performing a factor analysis, to test whether stressors and resources could best be explained by a single underlying factor. This was done through a principal components analysis with oblique rotation (because factors were expected to correlate), which retained all factors with an eigenvalue greater than 1.

**Figure 1:**
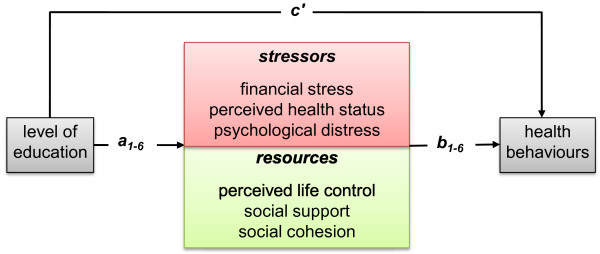
**Multiple stressors and resources mediate the education-health behaviour link**.

The mediation of the education-health behaviour relations by the stressors and resources was tested directly with a bootstrapping method for multiple mediator models (Preacher & Hayes, 2008). This method allowed all mediational paths of the various stressors and resources to be included simultaneously in one model, and this was done separately for each health behaviour. The bootstrapping method yields a point estimate and a 95% confidence interval for each indirect (i.e., mediation) effect a*b in the model (see Figure 1), while c' represents the direct effect of × (i.e., educational level) on Y (i.e., health behaviours) that is independent of the pathways through the mediators (i.e., stressors and resources). The total effect of × on Y, represented by coefficient c, is thus comprised of direct effect c' and all indirect effects

a_1-6_*b_1-6_. When the confidence interval for the indirect effect does not contain zero, the indirect effect is significant. The sampling distribution of the product term a*b is almost always skewed and bootstrapping is a method that involves a nonparametric resampling procedure to generate an empirical approximation of the sampling distribution of a*b, and thereby prevent the loss of statistical power. The number of bootstrap resamples was set to 5000, indicating that 5000 samples (with replacement) were taken from the data set to calculate a value for each mediation effect (Preacher & Hayes, 2008). The third research objective was accomplished by averaging all the separate measures of stressors and resources into one overall measure. This overall Stressors & Resources measure was entered as a single mediator in a separate model for each health behaviour.

All relations with education were controlled for possible confounding, by including demographic variables (i.e. gender, age, ethnicity, and neighbourhood of residence) and chronic disease status (since chronic diseases may impact health behaviour through routes other than stress, i.e. physical impairment) as covariates in all analyses. Alpha level for tests of significance was set a priori at *p *= .05. We used PASW statistical software version 19.0 (SPSS Inc., Chicago, USA) for all analyses.

### Ethical considerations

Data for this study were collected by the Municipal Health Service Utrecht for purposes of public health promotion. The research was carried out according to national guidelines for survey research among the adult population. Data collection procedures assured confidentiality by the use of self-administered, anonymous questionnaires. Ethical approval was not required as the study was voluntary and confidentiality was fully guaranteed.

## Results

From our sample of 3497 respondents, a total of 447 (12.8%) respondents were excluded from the analyses because they had missing data on educational level (*n *= 96), had missing data on one or more health behaviours (*n *= 264), or on one or more of the mediators (*n *= 249; these categories were not mutually exclusive). Results from a logistic regression showed that higher age, lower level of education, and a non-Western background (but not gender) was related to having missing data. The final sample counted 3050 respondents with complete data. The mean age of the sample was 44.9 years (SD = 15.9) and 56.3% were female (n = 1718). The majority was of Western descent (87.2%). The percentage of people with no education or primary school only was 10.9% (n = 332), 23.3% (n = 712) finished lower vocational school to intermediate secondary school, 19.0% (n = 579) intermediate vocational to higher secondary school, and 46.8% (n = 1427) received higher vocational to university education. Other descriptives are presented in Table 1.

**Table 1:** Sample characteristics (*N *= 3050).

Variable		*N *(%)	Mean (SD)	Range
Age			44.9 (15.9)	17-96
Male		1332 (43.7)		
Level of education	*no education and primary school*	332 (10.9)		
	*lower vocational/intermediate secondary school*	712 (23.3)		
	*intermediate vocational/higher secondary school*	579 (19.0)		
	*higher vocational school/university*	1427 (46.8)		
Non-Western background		389 (12.8)		
Chronic diseases**^a^**	*Cardiovascular disease*	460 (15.1)		
	*Musculoskeletal disorder*	775 (25.4)		
	*Diabetes*	185 (6.1)		
	*Lung diseases*	268 (8.8)		
	*Cancer*	56 (1.8)		
Stressors	Financial stress		1.6 (0.5)	1-3
	Perceived health status		1.8 (0.5)	1-3
	Psychological distress		1.3 (0.3)	1-3
Resources	Perceived life control		2.5 (0.4)	1-3
	Social support		2.7 (0.4)	1-3
	Social cohesion		2.3 (0.4)	1-3
Health behaviours	Exercise (minutes per week)		107.8(156.6)	0-750
	Vegetable consumption (daily no. of serving spoons)		3.6 (1.9)	0-12
	Breakfast frequency (per week)		5.9 (2.1)	0-7
	Smoking (cigarettes/cigars per day)		3.0 (6.8)	0-30

**^a ^**number of respondents indicating that they currently suffered, or had suffered during the last twelve months, the disease.

We first explored the data through examining correlations between educational level, stressors and resources and the four health behaviours. All expected relations were observed, namely that level of education was correlated with the four health behaviours (range *r *= .14 to -.17, all *p*'s < .01); education was positively correlated with the three resources (range *r *= .10 to .29, all *p *values < .01) and negatively to all three stressors (range *r *= -.20 to -.36, all *p*'s < .01); and higher levels of stressors were associated with more risky health behaviours (range *r *= -.05 to -.21 all *p*'s < .01), and vice versa for resources (range *r *= .07 to .19, all *p*'s < .01), with the notable exception of social cohesion that did not correlate with exercise.

The stressors and resources intercorrelated in the expected direction (stressors positively, resources positively, stressors and resources negatively) from *r *= -.11 to *r *= -.64 (all *p*'s < .01), providing a first indication that stressors and resources tend to co-occur. Results from the factor analysis revealed only one factor with an eigenvalue greater than 1, which explained 47.4% of the total variance. Examination of the factor loadings (see Table 2) showed that all stressors and resources correlated strongly with this single factor, except for social cohesion, which showed a moderate correlation. These results indicate that the various stressors and resources co-occur within individuals, and can be represented by a single factor. This is further supported by the results from the mediation analysis.

**Table 2:** Correlation coefficients between scales and Factor 1

Scale	Correlation with Factor 1
Financial stress	.61

Perceived health status	.70

Psychological distress	.83

Perceived life control	-.81

Social support	-.73

Social cohesion	-.36

### Mediation by stressors and resources

Looking at the *a *weights in the bootstrap analyses (Table 3), it is evident that level of education was negatively associated with all three stressors (range *B *= -0.04 to -0.16, all *p*'s < .01) and positively with all three resources (range *B *= 0.05 to 0.09, all *p*'s < .01) for all four behaviours while controlling for other demographic variables and chronic disease. This again indicates that higher levels of stressors and lower level of resources indeed co-occur among the lower educated. With regard to the second objective, results showed that level of education was positively associated with all four health behaviours (*c *weights in Table 2), and continued to have a direct relationship (*c' *weights) with health behaviours in the presence of the mediators, with the exception of exercise. For all four health behaviours the relationship between education and health behaviour was partially mediated by three or more stressors and resources.

**Table 3:** Mediation by stressors and resources of the education-health behaviours relationships.

Dependent variable *Mediating variables *	Association between education and mediator (*a*)	Association between mediator and health behaviour (*b*)	Direct association (*c*')	Indirect association (a*b)	95% CI for a*b	Total association (*c*)
**Exercise**			4.90			11.81**
*Financial stress*	-0.12**	-17.65*		2.20	0.73, 3.77	
*Perceived health status*	-0.08**	-44.27**		3.36	2.05, 4.95	
*Psychological distress*	-0.04**	6.08		-0.23	-1.17, 0.62	
*Perceived control*	0.06**	11.00		0.61	-0.55, 1.85	
*Social support*	0.06**	16.26^+^		1.00	0.19, 1.94	
*Social cohesion*	0.05**	-0.38		-0.02	-0.74, 0.67	
**Vegetable consumption**			0.36**			0.41**
*Financial stress*	-0.12**	0.04		-0.01	-0.03, 0.01	
*Perceived health status*	-0.08**	-0.36**		0.03	0.01, 0.05	
*Psychological distress*	-0.04**	0.18		-0.01	-0.02, 0.00	
*Perceived control*	0.06**	0.33^+^		0.02	0.00, 0.04	
*Social support*	0.06**	0.15		0.01	0.00, 0.02	
*Social cohesion*	0.05**	0.23*		0.01	0.00, 0.02	
**Breakfast frequency**			0.26**			0.41**
*Financial stress*	-0.16**	-0.46**		0.08	0.05, 0.11	
*Perceived health status*	-0.11**	-0.30*		0.03	0.01, 0.06	
*Psychological distress*	-0.06**	0.07		0.00	-0.03, 0.02	
*Perceived control*	0.08**	-0.29^+^		-0.02	-0.05, 0.00	
*Social support*	0.09**	0.68**		0.06	0.04, 0.08	
*Social cohesion *	0.05**	0.17		0.01	0.00, 0.02	
**Smoking**			-1.14**			-1.45**
*Financial stress*	-0.12**	1.74**		-0.22	-0.32, -0.14	
*Perceived health status*	-0.08**	1.06*		-0.08	-0.14, -0.03	
*Psychological distress*	-0.04**	0.52		-0.02	-0.07, 0.03	
*Perceived control *	0.06**	1.33*		0.07	0.02, 0.14	
*Social support*	0.06**	-0.89^+^		-0.05	-0.12, -0.01	
*Social cohesion *	0.05**	-0.16		-0.01	-0.03, 0.02	

^+ p < .05; * *p *< .01; ** *p *< .001^

^All analyses were controlled for age, gender, ethnicity, neighbourhood of residence, and chronic disease status.^

To illustrate the results displayed in Table 3, the results of physical exercise are discussed in more detail. The total association of education with physical exercise is *B *= 11.81 (*p *< .001), meaning that one level increase in educational attainment is associated with almost 12 more minutes exercise per week. Of this association, little over 2 minutes is mediated by financial stress (a*b weight, *B *= 2.20; 95% confidence interval [CI] = 0.73, 3.77), over 3 minutes by perceived health status (B a*b path = 3.36; 95% CI 2.05, 4.95) and 1 minute by social support (a*b weight, *B *= 1.00; 95% CI 0.19, 1.94). The other mediators are not significant (i.e., the confidence interval contains '0'). This means that about half of the relation between education and health behaviour can be explained through these mediators, leaving the direct relation of education with exercise not significant in the presence of resources and stressors (*c' *weight*, B *= 4.90, *p *= .13).

Although these mediation effects might not sound too spectacular, primarily because -contrary to what one would expect based on the literature on socioeconomic health disparities- the relation of education with the health behaviours is modest, the associations between health behaviours and the resources and stressors are notable. For example, the *b *weight from perceived health status to exercise is *B *= -44.27 (*p *< .001), indicating that a one-point increase (indicating worse health) is associated with 44 minutes less exercise per week (since the analysis is controlled for chronic disease, this association is unlikely to reflect physical disability). A 1-point increase in financial stress equals an additional 17 minutes in exercise. Hence, although stressors and resources co-occur among the lower educated, it seems that independent of educational level the direct associations between health behaviours and the stressors and resources are large relative to the total effect of education.

Note that psychological distress was not a significant mediator for any of the health behaviours when controlling for confounders and the other mediators in the model. Social cohesion only mediated the association between education and vegetable consumption. Furthermore, perceived life control is a significant mediator for vegetable consumption and smoking. In contrast to expectations and the univariate correlations, it is associated with more smoking. However, because all the mediating variables were to some extent correlated, entering them all in the same model, could have resulted in over-adjustment. All analyses were therefore repeated with all stressors and resources entered as a single mediator for all four health behaviours (data not shown). As opposed to the results from the full models, psychological distress was now a significant mediator for all four health behaviours. Perceived life control as a single mediator was no longer significant for smoking, but now showed a positive association with exercise and breakfast frequency. Finally, social support was a significant single mediator for vegetable consumption, while social cohesion became significant for breakfast frequency and smoking.

Finally, since it has been suggested that the aggregate effects of stressors and resources is stronger than the sum of the individual effects [28], and the factor analysis confirmed that a single factor best explains the different stressors and resources measures, all six separate measures were averaged into a single measure of Stressors and Resources. This overall measure was entered as a single mediator into the bootstrap analyses. Results revealed (Table 4) that the mediational relations of the separate mediators were comparable with the mediational relations of the overall measure for all four health behaviours. Although the *B *values of the direct associations of the overall measure with the health behaviours appear to be somewhat stronger, the overall measure has a smaller range than the individual stressors and mediators. As a result, direct and indirect effects of the overall Stressors & Resources measure are of similar size as the direct and indirect effects of the significant individual stressors and resources added together.

**Table 4:** Mediation of the education-health behaviours relationships by the overall Stressors & Resources measure.

Dependent variable	Association between education and mediator (*a*)	Association between mediator and health behaviour (*b*)	Direct association (*c*')	Indirect association (a*b)	95% CI for a*b	Total association (*c*)
Exercise	-0.05**	-108.33**	6.53	5.29	3.75, 7.12	11.81**

Vegetable consumption	-0.05**	-0.89**	0.37**	0.04	0.03, 0.06	0.41**

Breakfast frequency	-0.07**	-2.06**	0.27**	0.14	0.11, 0.17	0.41**

Smoking	-0.05**	5.41**	-1.18**	-0.26	-0.36, -0.19	-1.45**

## Discussion

Recent studies indicate that differences in health behaviours largely account for the socioeconomic health disparities observed in a range of studies [e.g., [11-13,29]]. How exactly socioeconomic position translates into health behaviours is not that clear. It has been suggested that differences in health behaviours may, at least partially, stem from differences in stressors and psychosocial resources. Although some studies support this idea, it remains to be examined whether stressors and resources co-occur among the lower educated, simultaneously impact health behaviours, mediate the relation between education and behaviour, and whether co-occurring stressors and resources are better examined separately or in one or two overall measures [28,41]. The aim of the present study was to investigate these issues.

The current study revealed that the presence of stressors and the absence of resources co-occur among those with lower educational levels. A lower education thus placed people at a disadvantaged position for all the stressors (i.e. financial stress, worse perceived health status and psychological distress) and resources (i.e. perceived life control, social support and social cohesion) examined here. Whereas the focus of our study was on the association of this accumulated disadvantage with health behaviours, it is important to note that exposure to stressors and having limited resources also have a direct negative impact on quality of life and health [23,49].

Subsequent analyses showed that, as others have previously observed [e.g.,[12,18,19]], higher educational level is associated with more exercise, a higher vegetable consumption and breakfast frequency, and less smoking. But most notably, as we proposed, stressors and resources were associated with health behaviours and partially mediated the association with education. Lower education was associated with higher exposure to stressors and less availability of resources, which, in turn, predicted less healthy behaviours irrespective of education.

Examining the mediation and direct associations of individual stressors and resources, different relations were observed for each of the health behaviours. The educational relation with vegetable consumption was mediated by perceived health status, perceived life control and social cohesion, while the relation with smoking and breakfast frequency was mediated by four out of the six stressors and resources, i.e. financial stress, perceived health status, perceived life control and social support. For exercise financial stress, perceived health status, and social support were significant mediators. Depending on the behaviour, financial stress and perceived health status were significant mediating stressors, and perceived life control and social support were significant mediating resources. Surprisingly however, perceived life control showed a negative relation with breakfast frequency and smoking (but not with vegetable consumption), and psychological distress was not a mediator for any of the health behaviours. However, when mediators are highly correlated, entering them together in the model may lead to suppression or over-adjustment of the effects of the single mediators. Therefore, all analyses were rerun with the single stressors and resources. The pattern of results was largely the same, with notable exceptions for psychological distress and perceived life control. Psychological distress was now a significant mediator for all four health behaviours. Perceived life control was no longer a significant mediator for smoking, but it did become a significant mediator for exercise and breakfast frequency, having positive associations with both behaviours. These deviations from the previous results could be a sign of suppression or over-adjustment in the full model, but findings are ambiguous. Taken together, the results from the mediation analysis suggest that level of education is predictive of the degree to which people experience financial, emotional and physical stressors, or accumulate perceived life control, social support and neighbourhood social cohesion, and that these stressors and resources explain - at least to some extent - how educational level is predictive of health behaviours.

When interpreting these results, it must be noted that, although it has been shown that unhealthy behaviours indeed co-occur among lower socioeconomic groups [17], and the combination of several less healthy behaviours add up to explain a large part of the socioeconomic health gap [12], the association of education with each of the health behaviours is modest. Another issue is that level of education remains associated with three of the four health behaviours when the Stressors and Resources are taken into account. Hence, the stressors and resources examined here do not offer a comprehensive explanation of the education-health behaviour link. Other variables that we did not measure may underlie the remaining direct relation with education, such as knowledge, awareness, social norms or health literacy [50-52].

In a final analysis, we found that combining stressors and resources in a single measure hardly changed the pattern of direct and indirect associations between education and health behaviours, as compared to using the individual variables. This confirmed the findings from the factor analysis and mediation analysis that stressors and lack of resources can be viewed as conceptually similar.

Besides their co-occurrence and mediation effects, a considerable *direct *relationship between stressors and resources, and health behaviours was observed. For example, by multiplying the *B *value of financial stress for exercise with the range of the financial stress scale (i.e. 2), it was found that the difference between a minimum and maximum score on the financial stress scale was associated with a reduction of approximately 35 minutes exercise per week, and an increase of 3.5 cigarettes per day. This observation implies that although stressors and resources cluster among lower educated people, part of their influence on behaviour is independent of educational attainment. Interventions directed at alleviating stressors and building psychosocial resources, like financial management, coping with chronic disease, or training of social skills may therefore have beneficial consequences for everyone, including those in disadvantaged groups who experience higher rates of difficulties. Interventions that aim to disproportionately reduce stressors and resources among the lower educated may relieve some of the inequalities in health behaviours, but this is limited to the extent stressors and resources explain these inequalities. This impact may be considerably larger, and easier to accomplish, compared to interventions to promote socioeconomic position. Although by no means we mean to imply that measures to decrease socioeconomic inequalities have no effect on health inequalities, studies indicate that with smaller income inequalities, health behaviours may become even more important in determining health inequalities [4,12]. That is why we propose that measures to narrow structural inequalities should be accompanied by health communication programs that address psychological and behavioural factors in disadvantaged groups.

### Strengths and Limitations

The strengths of the present study are the use of reliable and valid measures in a large sample and the testing of relations across multiple behaviours. A limitation is the response rate of 52.2%. In addition, 12.8% of the respondents eligible for the analysis had missing data, and having missing data was associated with lower education, older age, and having a non-Western background. This may indicate a selection bias, although recent studies suggest that lower response rates do not necessarily affect survey results [53,54]. In addition, people with lower education were still well represented (34.2% in our sample against approx. 30% for the population). With 12.8% in our sample against 21% in the population of Utrecht, however, respondents with a non-Western background were somewhat underrepresented. Although more than sufficient participants were available from all educational levels and ethnic background to conduct the analyses, there may be limitations in generalizability of the findings. Other limitations of this study are that the data are cross-sectional so that causal inferences cannot be made. It is possible, for example, that health behaviours influence people's level of stressors and resources. However, it is very likely that educational level preceded the other measures in this adult sample (mean age 44.9 years). Moreover, since the resources and stressors measured here are relatively stable factors [28,39], they are very likely to have preceded the behaviours measured in the week prior to the completion of this questionnaire. A second limitation is that behaviour was measured subjectively and may therefore be subject to social desirability bias or memory impairments. Finally, averaging all the stressors and resources into one overall measure weights them all equally, although our results imply that some mediators are more meaningful than others.

## Conclusions

Stressors and lack of psychosocial resources accumulate among those with lower socioeconomic position, are related with health behaviours, and partially explain how lower education translates into less healthy behaviours. Although longitudinal studies are needed to clarify exactly how stressors and resources accumulate among the lower-educated and affect health behaviours, this study suggests that both stressors and resources could be relevant intervention targets for bridging the health gap between people with different socioeconomic backgrounds.

## Competing interests

The authors declare that they have no competing interests.

## Authors' contributions

BCM had full access to all of the study data and takes responsibility for the accuracy of the data analysis and interpretation; he formulated the research question, conducted data analysis and wrote the paper. MB provided guidance in the formulation of the research question, the data analysis and interpretation, and critically revised the manuscript. HS and EA were responsible for the construction of the questionnaire and the data collection, and commented on earlier versions of the manuscript. CMJW guided the formulation of the research question and critically revised the manuscript. All authors read and approved the final manuscript.

## Pre-publication history

The pre-publication history for this paper can be accessed here:

http://www.biomedcentral.com/1471-2458/11/798/prepub
